# First-principles calculations of high-pressure physical properties anisotropy for magnesite

**DOI:** 10.1038/s41598-022-07705-3

**Published:** 2022-03-07

**Authors:** Zi-Jiang Liu, Xiao-Wei Sun, Cai-Rong Zhang, Shun-Jing Zhang, Zheng-Rong Zhang, Neng-Zhi Jin

**Affiliations:** 1 School of Mathematics and Physics, Lanzhou Jiaotong University, Lanzhou, 730070 China; 2 School of Electronic Engineering, Lanzhou City University, Lanzhou, 730070 China; 3 Department of Applied Physics, Lanzhou University of Technology, Lanzhou, 730050 China; 4 School of Information Engineering, Lanzhou City University, Lanzhou, 730070 China; 5 Key Laboratory of Cloud Computing of Gansu Province, Gansu Computing Center, Lanzhou, 730030 China

**Keywords:** Planetary science, Solid Earth sciences

## Abstract

The first-principles calculations based on density functional theory with projector-augmented wave are used to study the anisotropy of elastic modulus, mechanical hardness, minimum thermal conductivity, acoustic velocity and thermal expansion of magnesite (MgCO_3_) under deep mantle pressure. The calculation results of the phase transition pressure, equation of state, elastic constants, elastic moduli, elastic wave velocities and thermal expansion coefficient are consistent with those determined experimentally. The research results show that the elastic moduli have strong anisotropy, the mechanical hardness gradually softens with increasing pressure, the conduction velocity of heat in the [100] direction is faster than that in the [001] direction, the plane wave velocity anisotropy first increases and then gradually decreases with increasing pressure, and the shear wave velocity anisotropy increases with the increase of pressure, the thermal expansion in the [100] direction is greater than that in the [001] direction. The research results are of great significance to people’s understanding of the high-pressure physical properties of carbonates in the deep mantle.

## Introduction

Magnesite is a likely main host of carbonates in the mantle and plays an important role in the transport and storage of carbon in the Earth's mantle. Its high-pressure physical properties are crucial for understanding the deep carbon cycle^1^. However, the structure of its high-pressure phase and its phase transition boundary are controversial. The experiment shows that the phase transition pressure ranges from magnesite (space group R$\overline{3}$c) to magnesite-II (space group C2/m) is 75–115 GPa^2–6^, while the theoretical result is 75–101 GPa^7–13^.

The elastic properties of minerals control the stress–strain relationship under elastic loading and are related to understanding strength, hardness, brittle/ductile behavior, damage tolerance, and mechanical stability. The elastic modulus controls the propagation of elastic waves, including the seismic anisotropy of the crust and mantle, so it is very important for the interpretation of seismic data. As derivatives of the free energy, they are also related to the thermodynamic properties of minerals and are important for understanding the equation of state, phase stability and phase transition mechanism^14^. However, it is very difficult to measure the elastic constant under high temperature and high pressure. Recently, the elastic constants of magnesite are measured only up to 13.7 GPa^15^. The available results of the elastic properties are mainly limited to first-principles calculations^1,9,11,16^, these studies mainly discuss the elastic properties and the elastic wave velocity of magnesite. The thermal expansion coefficient of magnesite is mainly measured at low pressure, while the results under high pressure and high temperature are extrapolated^17–19^, and the result is also obtained by theoretical calculation^1,20^. So far, the thermal expansion anisotropy of magnesite has not been reported. In addition, its hardness and minimum thermal conductivity anisotropy have not been studied.

In present work, the elastic properties, hardness, thermal conductivity, elastic wave velocity and thermodynamic properties of magnesite under high pressure are investigated using the first-principles calculations based on density functional theory with generalized gradient approximation (GGA) combined with the quasi-harmonic approximate Debye model. The calculated elastic constants, elastic wave velocity and volumetric thermal expansion coefficient of magnesite are in agreement with the results with the existing experimental data. On this basis, we study the anisotropy of the elastic modulus, mechanical hardness, minimum thermal conductivity, elastic wave velocity and linear thermal expansion coefficient of magnesite.

## Results and discussion

### Phase transition, structural parameters and equation of state

**Figure 1:**
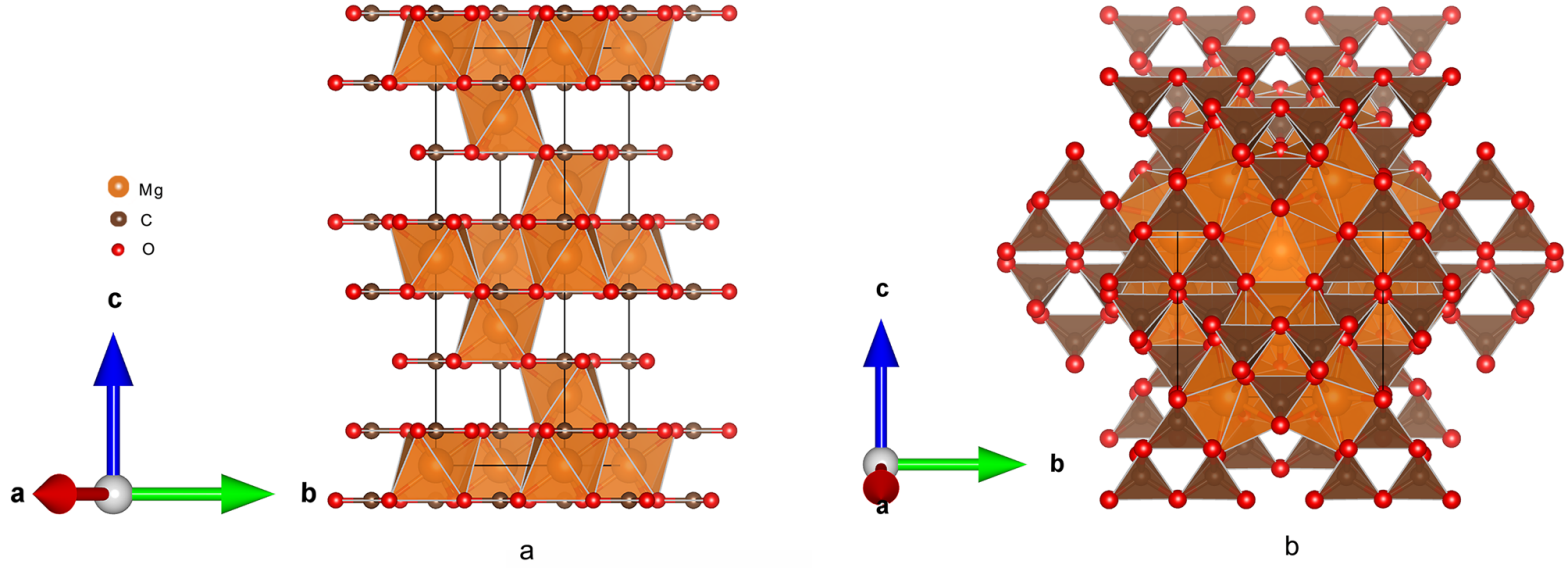
Crystal structures of magnesite (**a**) and magnesite-II (**b**).

The crystal structures of magnesite with space groups R$\overline{3}$c (Z = 6 formula units) and magnesite-II with space group C2/m (Z = 12 formula units) are shown in Fig. 1, respectively. The calculated phase transition pressure from magnesite to magnesite-II is 72 GPa by using gibbs2 program^21^. This result is consistent with the recent experimental^6^ and theoretical^11^ results of 75 GPa. Therefore, the present work only studies the anisotropy of the physical properties for magnesite when the pressure rises to 80 GPa.

**Figure 2:**
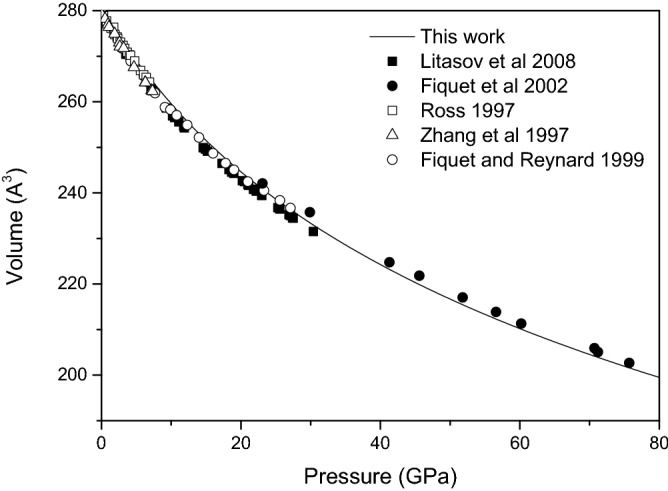
Equation of state for magnesite from 0 to 80 GPa.

As seen in Fig. 1, Magnesite has a hexagonal crystal system with rhombohedral symmetry and space group R$\overline{3}$c. The structural parameters, isothermal bulk modulus and equation of state for magnesite are determined from a third order Birch–Murnaghan equation of state^22^. Table 1 shows the present calculated structural parameters and isothermal bulk modulus of magnesite, along with the experimental data^19,23,24^. It is found that the present calculated results are in good agreement with previously reported values. The equation of state provides important information about minerals, which helps to model the composition of the deep layers of the earth. From Fig. 2, the present calculated equation of state from 0 to 80 GPa agrees well with the previous experimental data^17,19,23–25^. The agreement of present calculated structural parameters and equation of state with the experiment indicates the feasibility and reliability of the computational method.

**Table 1:** Calculated structural parameters of magnesite along with the experimental data.

	a (Å)	c (Å)	V (Å^3^)	K_0_	K_0_^′^
Present work	4.649	14.906	279.23	108.27	4.58
**Experimental results**					
Fiquet and Reynard^23^	4.628	15.055	279.14	108	4.6
Ross^24^	4.634	15.018	279.28	111	4 (fixed)
Zhang et al.^19^	4.635	15.013	279.32	103	4 (fixed)

### Single-crystal elastic constants

The elastic properties of the earth’s minerals are crucial to understanding their internal properties, especially in terms of their chemical composition and the propagation of seismic acoustic waves. Magnesite has six ($c_{11} , \, c_{12} , \, c_{13} , \, c_{14} , \, c_{33} , \, c_{44}$) independent elastic constants since $c_{66} = {{\left( {c_{11} - c_{12} } \right)} \mathord{\left/ {\vphantom {{\left( {c_{11} - c_{12} } \right)} 2}} \right. \kern-\nulldelimiterspace} 2}$. In order to confirm its mechanical stability, the following mechanical stability criteria are checked^26^:

1 $$c_{11} > \left| {c_{12} } \right|,c_{44} > 0,2c_{13}^{2} < c_{33} (c_{11} + c_{12} ),2c_{14}^{2} < c_{44} (c_{11} - c_{12} ).$$

In this work, all the calculated elastic stiffness constants $c_{ij}$ satisfy the mechanical stability criteria, so it may be said that magnesite is mechanically stable.

**Figure 3:**
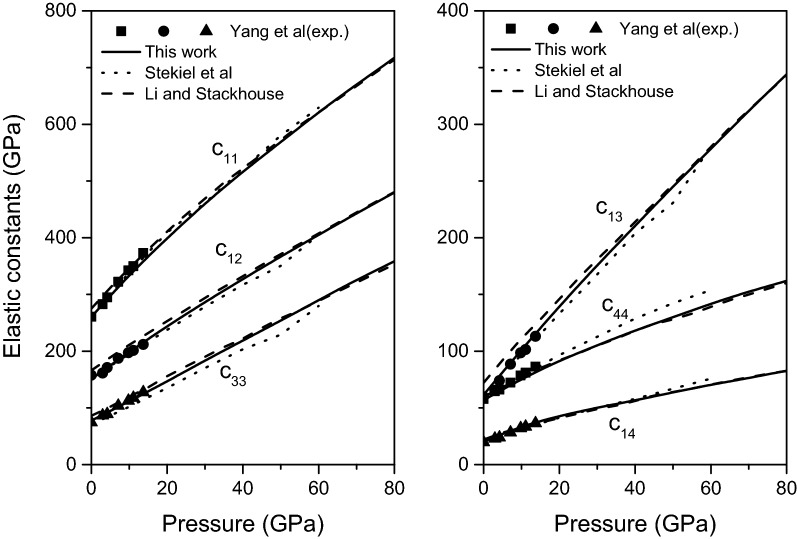
Elastic constants of magnesite from 0 to 80 GPa.

The calculated elastic constants of magnesite from 0 to 80 GPa are plotted in Fig. 3 and the data at 0 GPa are summarized in Table 2, compared with the previous experimental^15^ and theoretical^11,16^ results. It can be clearly seen from Fig. 3 and Table 2 that the present calculated elastic constants of magnesite are in excellent agreement with the available experimental and theoretical results, and gradually increase with the pressure.

**Table 2:** Calculated elastic constants ($c_{ij}$, in GPa) of magnesite, compared with the experimental and previous theoretical results at 0 GPa.

	$c_{11}$	$c_{12}$	$c_{13}$	$c_{14}$	$c_{33}$	$c_{44}$
Present work	260.38	77.45	61.55	20.21	150.85	56.81
Experimental results^15^	260.7	74.3	59.7	19.7	157.6	57.8
**Other theoretical results**						
Li and Stackhouse^11^	275	86	72	22	166	60
Stekiel et al.^16^	259.8	70.7	59.6	19.7	152.6	57.7

### Anisotropy of elastic modulus

The polycrystalline elastic moduli, such as bulk modulus $B$, shear modulus $G$ and Young’s modulus $E$, can be evaluated by Voigt–Reuss–Hill scheme^27–29^. For rhombohedral magnesite, bulk modulus $B$, shear modulus $G$ can be calculated from the Voigt bounds ($B_{V}$ and $G_{V}$) and Reuss bounds($B_{R}$ and $G_{R}$) from the following expressions:

2 $$B_{V} = \frac{{2c_{11} + c_{33} + 2c_{12} + 4c_{13} }}{9}$$

3 $$G_{V} = \frac{{\left( {2c_{11} + c_{33} } \right) - \left( {c_{12} + 2c_{13} } \right) + 3\left( {2c_{44} + {{\left( {c_{11} - c_{12} } \right)} \mathord{\left/ {\vphantom {{\left( {c_{11} - c_{12} } \right)} 2}} \right. \kern-\nulldelimiterspace} 2}} \right)}}{5}$$

4 $$B_{R} = \frac{1}{{\left( {2s_{11} + s_{33} } \right) + 2\left( {s_{12} + 2s_{13} } \right)}}$$

5 $$G_{R} = \frac{15}{{4\left( {2s_{11} + s_{33} } \right) - 4\left( {s_{12} + 2s_{13} } \right) + 3\left( {2s_{44} + s_{66} } \right)}}$$

6 $$B = \frac{{B_{V} + B_{R} }}{2},\;G = \frac{{G_{V} + G_{R} }}{2}$$

According to the bulk modulus $B$ and shear modulus $G$, Young's modulus $E$ is defined as $E = {{9BG} \mathord{\left/ {\vphantom {{9BG} {\left( {3B + G} \right)}}} \right. \kern-\nulldelimiterspace} {\left( {3B + G} \right)}}$.

**Figure 4:**
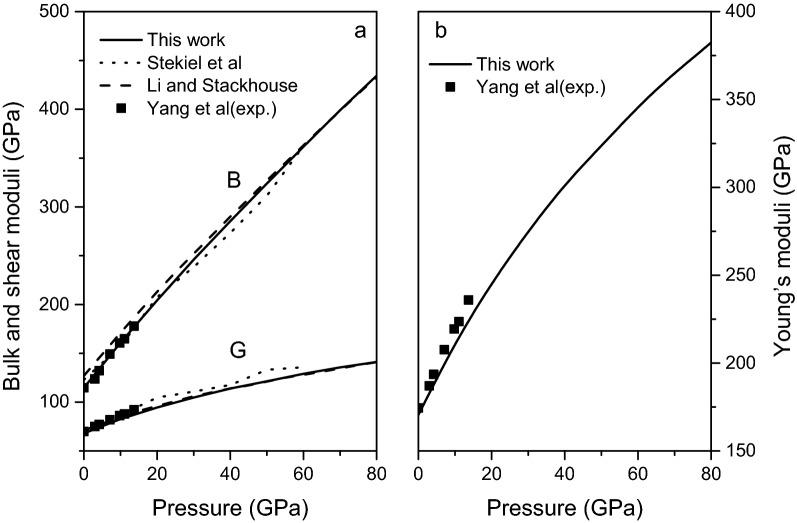
Bulk modulus $B$, shear modulus $G$ (**a**) and Young’s modulus $E$ (**b**) of magnesite from 0 to 80 GPa.

Figure 4 presents the changes of bulk modulus $B$, shear modulus $G$, and Young’s modulus $E$ of magnesite along with the previous experimental^15^ and theoretical^11,16^ results with pressure. As shown in figures, the present calculated elastic moduli increase smoothly and monotonically with increasing pressure, which agree well with the experimental and theoretical data.

The elastic anisotropy in mineral is of great significance due to its implication in geoscience as well as in crystal physics. In order to evaluate the elastic anisotropy of magnesite, Ranganathan and Ostoja-Starzewski universal anisotropy index^30^, Kube’s log-Euclidean anisotropy index^31^, and Chung and Buessem percent elastic anisotropy^32^ are used. The $A^{U}$,$A^{L}$,$A_{B}$, and $A_{G}$ are given by the following relations:

7 $$A^{{^{{_{U} }} }} = \frac{{B_{V} }}{{B_{R} }} + \, 5\frac{{G_{V} }}{{G_{R} }} - 6$$

8 $$A^{L} = \sqrt {\left[ {\ln \left( {\frac{{B_{V} }}{{B_{R} }}} \right)} \right]^{2} + 5\left[ {\ln \left( {\frac{{G_{V} }}{{G_{R} }}} \right)} \right]^{2} }$$

9 $$A_{B} = \frac{{B_{V} - B_{R} }}{{B_{V} + B_{R} }},\;A_{G} = \frac{{G_{V} - G_{R} }}{{G_{V} + G_{R} }}$$

**Figure 5:**
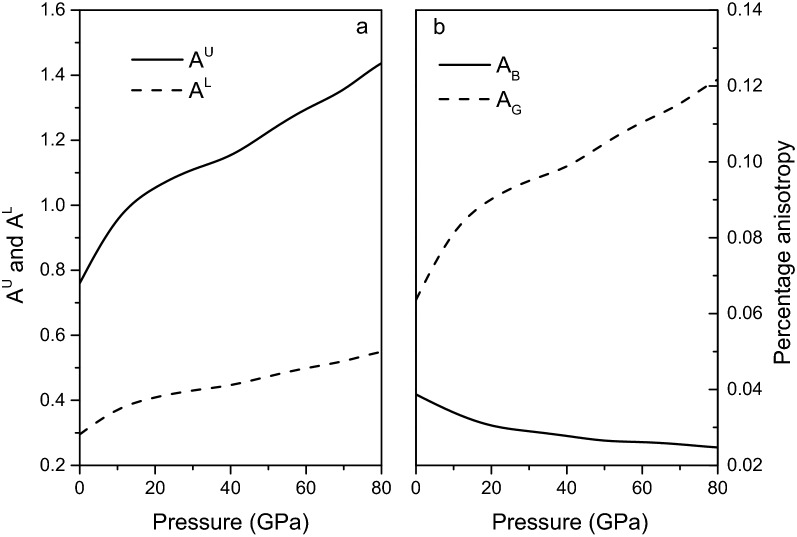
Universal anisotropy $A^{U}$, log-Euclidean anisotropy $A^{L}$ (**a**), and percentage of bulk ($A_{B}$) and shear ($A_{G}$) anisotropies (**b**) for magnesite from 0 to 80 GPa.

For an elastically isotropic crystal, $A^{U} = A^{L} = A_{B} = A_{G} = 0$, while the larger values of $A^{U}$, $A^{L}$, $A_{B}$ and $A_{G}$ represent a more elastic anisotropy. The universal anisotropy, log-Euclidean anisotropy, and percentage of bulk and shear anisotropies for magnesite are plotted in Fig. 5. From Fig. 5a may be observed that $A^{U}$ and $A^{L}$ increase with the increase of pressure, and the change trend is basically the same. It is found in Fig. 5b that the percentage of shear anisotropy increases with the increase of pressure, and the percentage of bulk anisotropy decreases, and the increase in the percentage of shear anisotropy is much greater than the decrease in the percentage of bulk anisotropy, this means that the contribution of shear anisotropy in the elastic anisotropy of magnesite is greater than that of bulk anisotropy.

**Figure 6:**
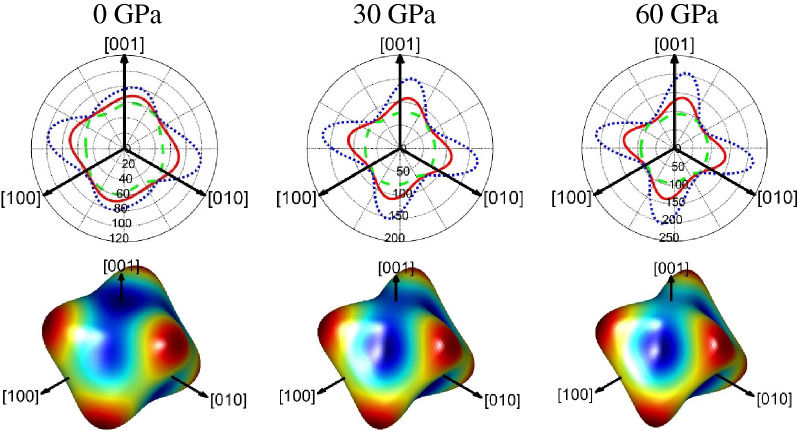
3D spatial distribution and its projection of shear modulus for magnesite at various pressures.

**Figure 7:**
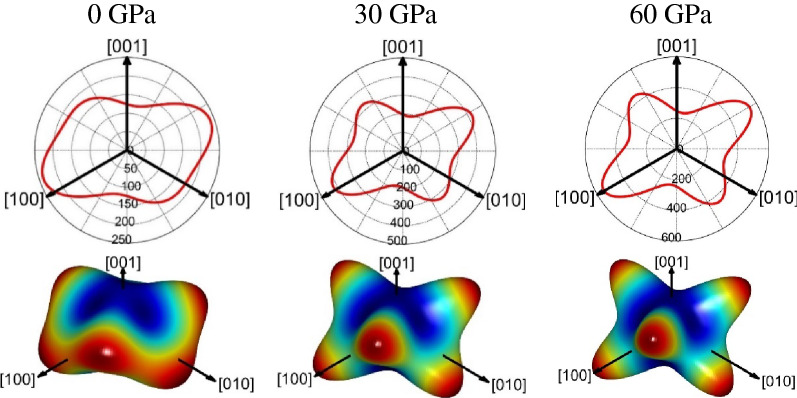
3D spatial distribution and its projection of Young’s modulus for magnesite at various pressures.

In order to furthermore elucidate this anisotropic behavior, the most straightforward method is to plot the three-dimensional contours of mechanical moduli. The direction dependent shear modulus ($G$) and Young’s modulus ($E$) for rhombohedral crystals can be defined as:

10 $$\frac{1}{G} = s_{44} \left( {\left( {s_{11} - s_{12} } \right) - \frac{1}{2}s_{44} } \right)\left( {1 - l_{3}^{2} } \right) + 2\left( {s_{11} + s_{33} - 2s_{13} - s_{44} } \right)l_{3}^{2} \left( {1 - l_{3}^{2} } \right)$$

11 $$\frac{1}{E} = s_{11} \left( {1 - l_{3}^{2} } \right)^{2} + s_{33} l_{3}^{4} + \left( {2s_{13} + s_{44} } \right)l_{3}^{2} \left( {1 - l_{3}^{2} } \right)$$

where $s_{ij}$ are the usual elastic compliance constants and $l_{1}$, $l_{2}$, and $l_{3}$ are the direction cosines in any arbitrary direction. The ElasticPOST program^33,34^ is used to obtain the 3D spatial distribution and their projection of shear modulus and Young’s modulus for magnesite at various pressures, and the results are displayed in Figs. 6 and 7, respectively. As can be seen, the 3D figures of shear modulus and Young’s modulus reveals a large degree of deviation in shape from the sphere. This means that magnesite has a strong anisotropy, which also confirms the calculation results in Fig. 5. The comparative analysis of shear modulus and Young’s modulus for different directions as seen from the planar projections also indicates the anisotropy level.

### Anisotropy of mechanical hardness

Vickers hardness is a fundamental property that is essential to describe the mechanical behavior of mineral, various semi-empirical relations have been proposed to estimate hardness using the elastic moduli. Vickers hardness is predicted using two theoretical models of hardness:

Chen’s model^35^:

12 $$H_{V}^{Chen} = 2\left( {k^{2} G} \right)^{0.585} - 3, \, \left( {k = {G \mathord{\left/ {\vphantom {G B}} \right. \kern-\nulldelimiterspace} B}} \right)$$

Tian’s model^36^:

13 $$H_{{_{V} }}^{Tian} = 0.92\left( \frac{G}{B} \right)^{1.137} G^{0.708}$$

**Figure 8:**
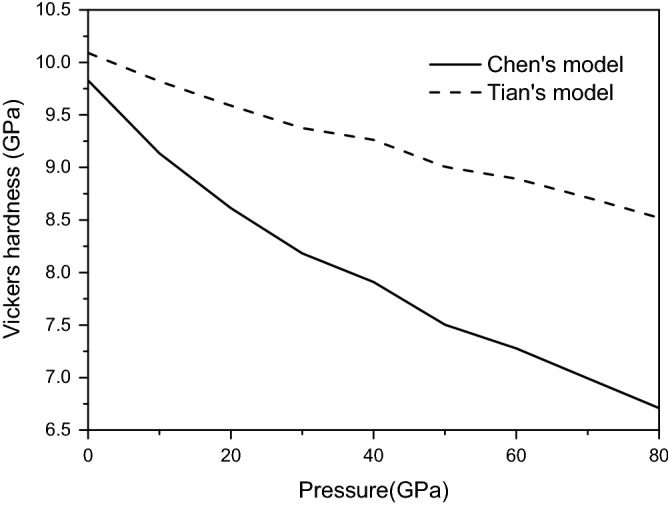
Vickers hardness of magnesite from 0 to 80 GPa.

**Figure 9:**
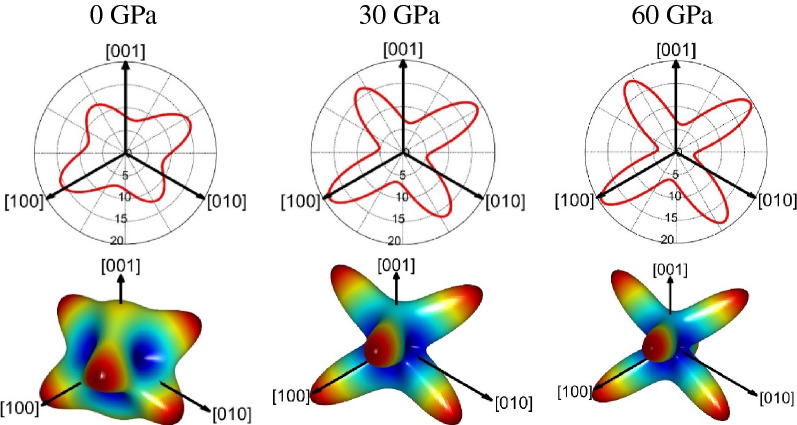
3D spatial distribution and its projection of hardness for magnesite at various pressures.

The calculated Vickers hardness of magnesite are depicted in Fig. 8 from 0 to 80 GPa. As illustrated in Fig. 8, the Vickers hardness decrease with increasing pressure, indicating magnesite becomes softer under high pressure, the Vickers hardness predicted by the Chen’s model is smaller than that Tian’s of the model in the entire pressure range. In order to evaluate the anisotropy of Vickers hardness of magnesite. The direction dependent hardness ($H$) can be obtained by fitting the direction dependent bulk modulus ($B$) and Young's modulus ($E$), defined as: $H = 0.130548175274347E^{2.2484942942017} B^{ - 1.51675853808829}$, where $B = {1 \mathord{\left/ {\vphantom {1 {\left( {\left( {s_{11} + s_{12} + s_{13} } \right) - \left( {s_{11} + s_{12} - s_{13} - s_{33} } \right)l_{3}^{2} } \right)}}} \right. \kern-\nulldelimiterspace} {\left( {\left( {s_{11} + s_{12} + s_{13} } \right) - \left( {s_{11} + s_{12} - s_{13} - s_{33} } \right)l_{3}^{2} } \right)}}$. The 3D spatial distribution and its projection of Vickers hardness for magnesite at various pressures are presented in Fig. 9. The Vickers hardness exhibit strong direction-dependent changes, resulting in large anisotropy, The 2D representations planar projection in different directions also show this result.

### Anisotropy of acoustic velocity

The velocities of plane and shear wave velocities of minerals can be calculated from the single crystal elastic constants. The plane wave velocity ($v_{P}$) and shear wave velocity ($v_{S}$) are calculated using^37^:

14 $$v_{P} = \sqrt {\frac{3B + 4G}{{3\rho }}} ,v_{S} = \sqrt {\frac{G}{\rho }}$$

**Figure 10:**
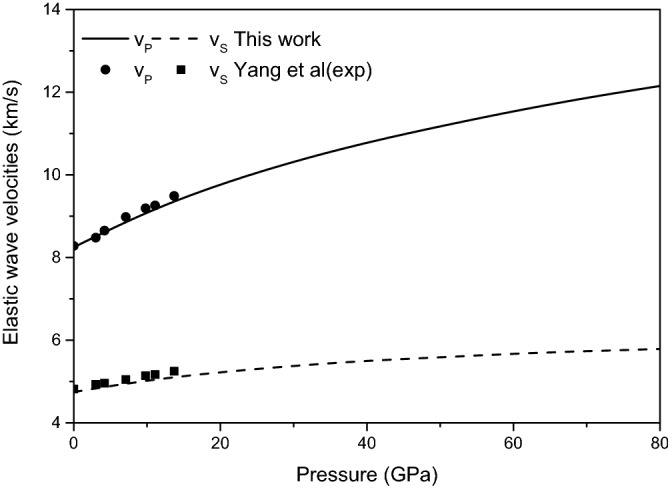
Elastic wave velocities of magnesite from 0 to 80 GPa.

The elastic wave velocities of magnesite are shown in Fig. 10 from 0 to 80 GPa. Figure 10 show that the calculated elastic wave velocity is in good agreement with the previous experimental results^15^ within the studied pressure range, the plane wave velocity $v_{P}$ propagate more speedily than the shear wave velocity $v_{S}$. The consistency between the calculated elastic wave velocity and the experimental results provides reliability for further research on elastic wave velocities anisotropy.

**Figure 11:**
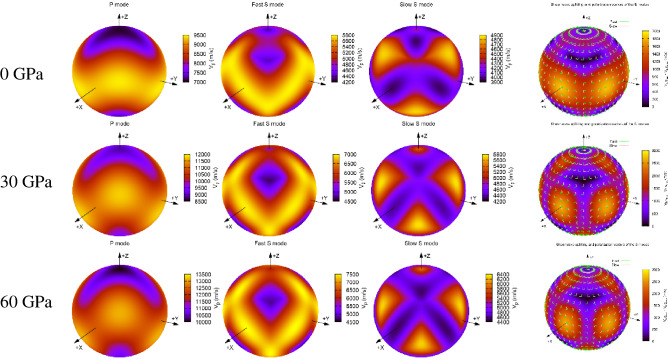
3D representation of the elastic wave velocities of the P-modes and S-modes (left) and the shear wave splitting and polarization vectors of the S-modes (right) for magnesite in different propagation directions at various pressures.

Directional elastic wave velocities are computed by solving Christoffel’s equation $\det \left| {C_{ijkl} n_{j} n_{l} - \rho v^{2} \delta_{ik} } \right| = 0$ ^38^, where $C_{ijkl}$ are the elastic stiffnesses, the $n_{j}$ are unit vectors of the wave propagation direction, $v$ is the acoustic velocity, and $\delta_{ik}$ is the Kronecker $\delta$. Using AWESoMe program^39,40^ with quadruple precision, the plane wave velocities and shear wave velocity and the shear wave splitting of magnesite in different propagation directions under various pressures are obtained, 3D representation of the elastic wave velocity and the shear wave splitting of magnesite are plotted in Fig. 11. It is observed from Fig. 11(left) that the plane wave velocities have minimum values along the z direction, firstly decreasing with the increase of pressure, and then gradually increasing. For the two shear wave velocity (fast and slow), the minimum values of the two wave velocities are shifted from the z direction yet they are still allocated around the z direction, but the magnitude of the shift gradually increases with the increase of pressure, especially the fast shear wave velocity, the results of shear wave splitting in Fig. 11(right) also further verify this result.

**Figure 12:**
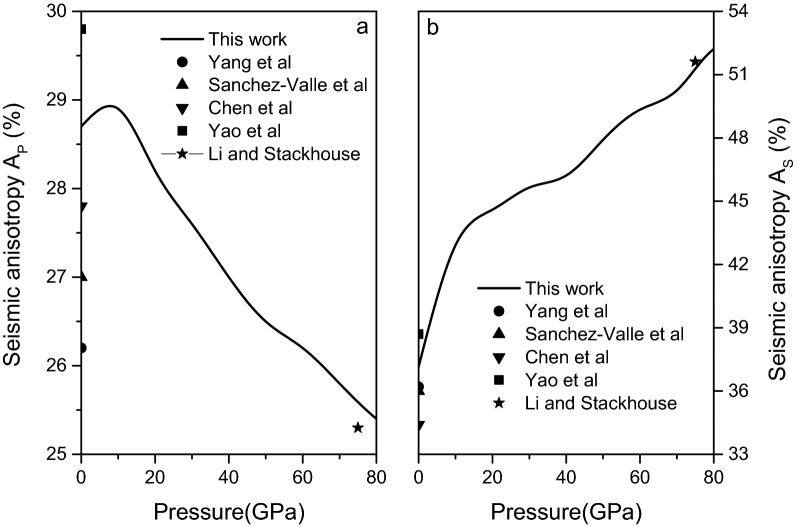
Anisotropy of plane (**a**) and shear (**b**) wave velocity of magnesite from 0 to 80 GPa.

Anisotropy of plane and shear wave velocity can be defined as $A_{P} = {{\left( {v_{P,\max } - v_{P,\min } } \right)} \mathord{\left/ {\vphantom {{\left( {v_{P,\max } - v_{P,\min } } \right)} {v_{P} }}} \right. \kern-\nulldelimiterspace} {v_{P} }} \times 100\%$ and $A_{S} = {{\left( {v_{S,\max } - v_{S,\min } } \right)} \mathord{\left/ {\vphantom {{\left( {v_{S,\max } - v_{S,\min } } \right)} {v_{S} }}} \right. \kern-\nulldelimiterspace} {v_{S} }} \times 100\%$, respectively. The calculated elastic wave velocities anisotropy of magnesite is presented in Fig. 12 and the data at 0 GPa are listed in Table 3, along with the previous experimental^15,41,42^ and theoretical^1,11^ results. It can be found from Table 3 that the maximum error between the calculated plane wave velocity anisotropy and the experimental^15^ value at 0 GPa is about 2.5%, and the maximum error between the shear wave velocity anisotropy and the experimental value^42^ is about 2.75%, indicating that the calculated data are in agreement with available experimental data. At low pressure, the plane wave velocity anisotropy increases with the increase in pressure, but gradually decreases at high pressure. However, the experimental result of Yang et al^15^ is that the plane wave velocity anisotropy increases with increasing pressure. The shear wave velocity anisotropy increases with increasing pressure, this result is consistent with the experimental^15^ and theoretical^1^ results. Especially at 75 GPa, the present calculated results are consistent with the theoretical results of Li and Stackhouse^11^.

**Table 3:** Plane wave velocity anisotropy $A_{P}$ and shear wave velocity anisotropy $A_{S}$ of magnesite at 0 GPa.

	$A_{P}$	$A_{S}$
Present work	0.287	0.3715
**Experimental results**		
Yang et al.^15^	0.260	0.362
Sanchez‐Valle et al.^41^	0.27	0.36
Chen et al.^42^	0.278	0.344
**Other theoretical results**		
Yao et al.^1^	0.298	0.387

### Anisotropy of minimum thermal conductivity

The thermal conductivity is a measure of material’s heat conduction ability. Generally, the thermal conductivity decreases to a limit value considered as the minimum thermal conductivity with increasing temperature. Therefore, it is of great significance to study the minimum thermal conductivity of magnesite. The minimum thermal conductivity of magnesite is calculated on the basis of Clark’s model^43^ and Cahill’s model^44^. In the Clarke model, the minimum thermal conductivity can be thought of as the limit the average phonon mean free path → the interatomic spacing. The Cahill model instead use a wavelength dependentmean free path to incorporate wave mechanics in the description of the average phonon mean free path. These models work well for many materials and give an intuitive description of the phonon limit of thermal conductivity.

Clark’s Model:

15 $$k_{min} = \, 0.87k_{B} M_{a}^{ - 2/3} E^{1/2} \rho^{1/6} ,\;M_{a} = M/(n \cdot N_{A} )$$

**Figure 13:**
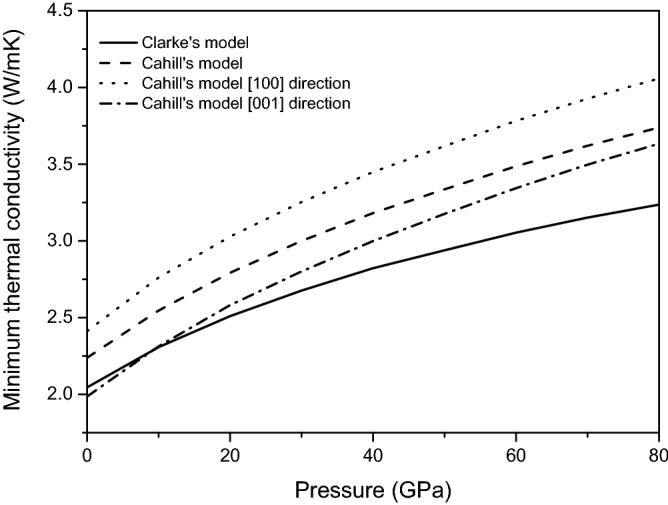
Minimum thermal conductivity of magnesite from 0 to 80 GPa.

Cahill’s Model:

16 $$k_{\min } = ({{k_{B} } \mathord{\left/ {\vphantom {{k_{B} } {2.48}}} \right. \kern-\nulldelimiterspace} {2.48}})n^{2/3} (v_{P} + 2v_{S} )$$

where $M_{a}$ is the average mass per atom, $E$ is Young’s modulus, $\rho$ is the density, $M$ is the molar mass, $n$ is the atomic number density per unit volume, $k_{B}$ is Boltzmann’s constant, $N_{A}$ is Avogadro’s number, respectively. Based on the two theoretical model, the calculated minimum thermal conductivity of magnesite from 0 to 80 GPa is shown in Fig. 13. It is seen that the minimum thermal conductivity of magnesite increases with the increase of the external pressure, the calculated results using the Cahill’s model is greater than that computed by the Clark’s model. This is due to the atom number density is considered in Cahill’s model, whereas the Clark’s model does not. Thus, the Clark’s model underestimates the thermal conductivity. That is, the data obtained by Cahill’s model should be closer to the real values than Clarke’s model.

To investigate the anisotropy of thermal conductivity, which can be summed from the plane wave velocities ($v_{P}$) and two shear wave velocity ($v_{S1}$ and $v_{S2}$). Therefore, the expression of Cahill's model can be changed in form as follows:

17 $$k_{min} = ({{k_{B} } \mathord{\left/ {\vphantom {{k_{B} } {2.48}}} \right. \kern-\nulldelimiterspace} {2.48}})n^{2/3} (v_{P} + v_{S1} + v_{S2} )$$

The calculated minimum thermal conductivities of magnesite in principal directions are also presented in Fig. 13, indicating the anisotropic characteristic of the minimum thermal conductivities. It can be observed that the kmin[100] values are always higher than the kmin[001] values within the pressure range of the study. It indicates that the conduction velocity of heat in the [100] direction is faster than that in the [001] direction. As can be seen from the crystallographic structures of rhombohedral magnesite, Mg, C and O atoms mainly align along the [100] direction.

### Anisotropy of thermal expansion

The thermal expansion coefficients and their temperature–pressure dependence are of importance in estimating the thermal properties of minerals. In present work, The Debye quasi-harmonic approximation (QHA) is used to calculate the thermal expansion coefficients of magnesite^45^. The volumetric thermal expansion coefficient ($\alpha_{V}$) can be obtained by the following expressions:

18 $$\alpha_{V} = \frac{{\gamma C_{V} }}{{B_{T} V}},\gamma = - \frac{{d\ln \theta_{D} }}{d\ln V}$$

where $\gamma$, $C_{V}$, $B_{T}$, $V$ and $\theta_{D}$ represent the thermal Grüneisen parameter, the heat capacities, the isothermal bulk modulus, the volume and the Debye temperature, respectively. The volume thermal expansion coefficient of magnesite at 300 K and 0 GPa is 3.376 × 10^–5^/K, in good agreement with the present calculated value of 3.688 × 10^–5^/K. Having obtained the volumetric thermal expansion at different temperatures and pressures, the thermal expansion along different directions can be calculated from the linear compressibility. For rhombohedral crystal, the expressions are as follows^46^:

19 $$\alpha_{V} = 2\alpha_{{\left[ {100} \right]}} + \alpha_{{\left[ {001} \right]}} ,\; \, {{\alpha_{{\left[ {100} \right]}} } \mathord{\left/ {\vphantom {{\alpha_{{\left[ {100} \right]}} } {\alpha_{{\left[ {001} \right]}} = {{K_{{\left[ {100} \right]}} } \mathord{\left/ {\vphantom {{K_{{\left[ {100} \right]}} } {K_{{\left[ {001} \right]}} }}} \right. \kern-\nulldelimiterspace} {K_{{\left[ {001} \right]}} }}}}} \right. \kern-\nulldelimiterspace} {\alpha_{{\left[ {001} \right]}} = {{K_{{\left[ {100} \right]}} } \mathord{\left/ {\vphantom {{K_{{\left[ {100} \right]}} } {K_{{\left[ {001} \right]}} }}} \right. \kern-\nulldelimiterspace} {K_{{\left[ {001} \right]}} }}}}$$

where $K_{{\left[ {100} \right]}}$ and $K_{{\left[ {001} \right]}}$ are the linear elastic compressibility in the [100] and [001] directions, respectively. it is obtained by^47^:

20 $$K_{[100]} = s_{11} + s_{12} + s_{13}, K_{[001]} = s_{33} + 2s_{13}$$

**Figure 14:**
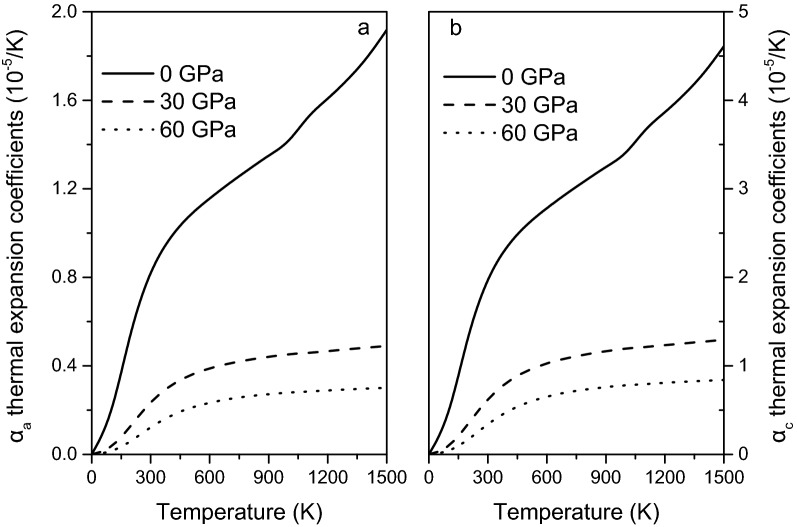
Linear thermal expansion coefficients $\alpha_{a}$ (**a**) and $\alpha_{c}$ (**b**) along the [100] = [010] and [001] directions of magnesite at various pressures.

The anisotropic linear thermal expansion coefficients of magnesite at various pressures are calculated and are depicted in Fig. 14. As can be seen, the thermal expansion in the [100] direction is the largest relative to the [001] directions in magnesite, and it decrease with increasing pressure. Unfortunately, there is no experimental data or theoretical calculation results to compare with the linear thermal expansion coefficient of magnesite. Thus, the present work is beneficial for future research on the thermal properties of minerals.

## Conclusions

The anisotropy of elastic modulus, mechanical hardness, minimum thermal conductivity, acoustic velocity and thermal expansion of magnesite under high pressure are investigated using the first-principles calculations within the density functional theory. The calculated phase transition pressure, equation of state, elastic constants, elastic moduli, elastic wave velocities and thermal expansion coefficient of magnesite are in excellent agreement with the previous experimental and theoretical results. It provides reliability for further research on the anisotropy of elastic modulus, mechanical hardness, minimum thermal conductivity, acoustic velocity and thermal expansion. The results of shear modulus and Young's modulus show that magnesite has strong anisotropy. The Vickers hardness changes strongly in different directions, leading to large anisotropy and softening under high pressure. Due to the higher probability of phonon collision in the [100] direction, the minimum thermal conductivity in the [100] direction is higher than that in the [001] direction and increases with the increase of pressure. The propagation of the plane wave along the z direction has a minimum value, which decreases first and then gradually increases as the pressure increases. The minimum value of the two shear wave velocities shifts from the z direction, and the magnitude of the shift gradually increases with the increase of pressure, especially in the fast S-mode. The plane wave velocity anisotropy first increases and then gradually decreases with increasing pressure, and the shear wave velocity anisotropy increases with the increase of pressure. As discussed in literature^1^, the elastic anisotropy of magnesite is much greater than that of the main minerals in the mantle, and its local enrichment provides a new explanation for the large local anisotropy in the transition zone. Finally, the anisotropy of thermal expansion is studied using the Debye quasi-harmonic approximation and elastic constants. It is found that the anisotropic linear thermal expansion coefficients in the [100] direction is the largest relative to the [001] directions and decrease with increasing pressure. The present work helps people to further understand the high-pressure physical properties of magnesite under deep mantle conditions, and also has important geophysical significance.

## Methods

First-principles calculations based on density functional theory^48,49^ are performed by using a Vienna Ab Initio Simulation Package (VASP)^50,51^ with the projector-augmented wave method (PAW)^52^. The Perdew-Burke-Ernzerhof revised for solids(PBEsol) in GGA^53^ is used to expound the exchange-correction function and calculate the self-consistent electronic density. The valence electron configurations are chosen 2*p*^6^3*s*^2^ for Mg, 2*s*^2^2*p*^2^ for C, and 2*s*^2^2*p*^4^ for O. Based on the results of plane-wave cutoff energy and k-mesh convergence tests, the cutoff energy for plane wave extension of the R$\overline{3}$c and C2/m structures for MgCO_3_ are set to 850 eV and 880 eV, and the Brillouin zone of Monkhorst–Pack grid sampling^54^ is 9 × 9 × 2 and 4 × 5 × 5, respectively. The convergence threshold for electronic self-consistent field and forces acting on the atoms are 1.0 × 10^−8^ eV and 0.02 eV/Å, respectively. The elastic constant is obtained using the stress–strain method^55,56^. The thermodynamic properties are calculated by the quasi-harmonic approximation (QHA) Debye approach^45^.
